# Belongingness and loneliness in higher education: a meta-analysis of pre- and post-pandemic trends

**DOI:** 10.3389/fpsyg.2025.1625957

**Published:** 2026-02-02

**Authors:** Gulsah Dost

**Affiliations:** School of Education, Durham University, Durham, United Kingdom

**Keywords:** COVID-19 pandemic, loneliness, belonging, belongingness, higher education, meta-analysis, student wellbeing, student mental health

## Abstract

**Introduction:**

This meta-analysis seeks to explore how the complex relationship between loneliness and belongingness in higher education students can be explained by a set of pre- and post-COVID-19 pandemic dynamics.

**Methods:**

A meta-analysis including 56 studies and involving a total of 30,062 participants was conducted, and the review explores direct relations and moderation through age, education, and country.

**Results:**

Results indicate a moderate-to-strong negative relationship between loneliness and belongingness (r = -0.48, 95% CI [-0.529, -0.422]), such that a consistent association was found across situations such that increases in one’s level of loneliness is associated with decreases in one’s level of belongingness. Nevertheless, there was no small-degree of inter-study heterogeneity (Q = 1058.86, *p <* 0.0001, I^2^ = 94.33%), which is a potential reason for the differences in the study populations and methods, employing a random-effects model to account for these discrepancies. After further scrutiny of the results, location, and year of study, and country did not moderate the effect size, which in turn reflects the stability of the association across context and time. In the subgroup analysis the effect size of the relationship between the level of the Information Technology (IT) usage and the externalisation was lower in the time of the pandemic than in the time preceding the pandemic. The effect size of the pre-pandemic group is -0.515 (95% CI: -0.589 to -0.441, *p <* 0.001 < 0.001) and the effect size of pandemic group is slightly smaller with -0.427 (95% CI: -0.502 to -0.352, *p <* 0.001 < 0.001). This means that although the level of loneliness have normalised, there have been a subtonic influence on perceived belonging of the novelty stressor caused by breakdowns in social connection from pandemic-level influences. In addition, no significant publication bias was observed.

**Discussion:**

Overall, these findings confirm the strong negative association between loneliness and sense of belonging and emphasise the important role in providing community support for students, especially during social disruptions.

## 1 Introduction

Students with a transition from high school to university in 2020 saw one of the most significant turning points in terms of their education pathway due to the rapid progression of COVID-19 to a pandemic on a global scale. As certain nations saw a rapid increase in the number of infected people, public health officials implemented vital measures to try and slow the virus’s spread. These strategies entailed mandatory containment of positive individuals, a prolonged period of broad self-isolation for symptomatic individuals and rigorous physical distancing procedures, all of which altered the way everyone acted (Ammar et al., 2020). This was not simply a matter of administration; these public health modifications created new spatial profiles that profoundly influenced the emotional and social experiences of relatives, friends, and nearby individuals. As a result of travel restrictions and adjustments to public health, it became virtually impossible for these pupils to attend schools located outside their home countries, thereby rendering it impossible for many students to attend their desired universities. When they could finally enroll, social distancing directions forbidding in-person lecturescaused a sudden increase in online learning. This massive upheaval left students without the traditional university experience of personal lectures, impromptu discussions, campus life (such as social clubs and extra activities) (Elumalai et al., 2021; Lederer et al., 2021; O'Connor et al., 2020). As a result, many students found themselves cut off from important opportunities to build meaningful relationships with faculty staff, department members and peers — relationships that typically develop through informal interaction in an academic environment (Porter et al., 2023). More than academic challenges, the results of these dramatic changes were feelings of loneliness and social disconnect that reached unprecedented level among students. Early research during the pandemic has demonstrated how widespread social wardens had increased loneliness, a process that appeared to be particularly pronounced for higher education students as they acclimatised to this novel environment (Groarke et al., 2020; Leal Filho et al., 2021). This period of self-isolation has shined a spotlight on the importance for all students to have access to robust support services and tools to work through their feelings around this major disruption in their education.

In the higher education context, Dost (2024a, 2024b) recently utilised structural equation modelling (SEM) with 284 female students and 120 male students. The researcher found that stress related to COVID-19 adversely affected both types of belongingness—collegiate community and degree department belonging —while concurrently elevating academic anxiety across diverse demographic groups. Loneliness emerged as a significant mediating variable, with pronounced effects observed among international and male students. Dingle et al. (2022) examined the impact of the COVID-19 pandemic on social connectedness and mental health of first-year students enrolled in a metropolitan university in Australia. The study involved 1,239 students (30.4% international) and used a 3 (cohorts: 2019, 2020, 2021) × 2 (enrolment status: domestic and international) between-group design. Results showed that both loneliness and university belonging were significantly worse during the first year of COVID-19 compared to the year before or after. Contrary to expectation, domestic students were lonelier than international students across all cohorts. The prevalence of mental health problems among United Kingdom university students is estimated to be around 20–25% (Lewis and Stiebahl, 2024; NHS England, 2023). However, since young populations are especially susceptible to mental health problems such as depression and anxiety during the COVID-19 pandemic (KCL, 2022), this probably underestimates current prevalence. Depressive and anxiety symptoms are particularly prevalent in the transition to university (Cage et al., 2021) and have been found to rise over the first year of university student life (Campbell et al., 2022). The proportion of home students (UK domiciled) who disclosed a mental health problem to their Higher Education Institutions (HEIs) rose from just under 1% in 2010/11 to 5.8% in 2022/23 (Lewis and Stiebahl, 2025). During the period of 2016/17 to 2022/23, the number of UK undergraduates who reported a mental health condition increased from 6 to 16%, meaning one in six students currently have a mental health problem (King’s College London (KCL) Report, 2023). It was reported by mental health charity Student Minds (2023) that 57% of students claimed that they had previously experienced a mental health issue and 27% that they had been diagnosed with a mental health condition (Farrier et al., 2024). Freshmen and minority students have been especially hit by these problems as highlighted by studies from various countries such as Italy (World Health Organization (2023), Germany (Jungmann and Witthöft, 2020) and the United States (Ettman et al., 2020) that signalled the enhanced risk those groups are exposed to. Furthermore, travel bans also had a negative influence on mental health, in particular for international students who had trouble beginning studies or returning home over break to visit their family and friends (Akiba et al., 2024). Establishing new relationships and a new social network is a significant concern of first year students in the undergraduate course as their friendships become crucial to their psychosocial adaptation to university life (Dost, 2024a). This may in turn erode an individual’s sense of social connectedness, and consequently the peer and faculty/department supports available to them (Burns et al., 2020). This sense of support is necessary to manage the challenges of a successful college or university transition and to feel connected to their university, college, or group.

Studies have focused on the effects of the COVID-19 pandemic on students’ sense of belonging and feelings of loneliness. For example, McCallum et al. (2021) reported on 1,217 participants aged 18 years or older who completed an online survey from March 28 to 31, 2020. Linear regression models revealed that COVID-19-related work and social adjustment difficulties, financial distress, loneliness, thwarted belongingness, eating a less healthy diet, poorer sleep and being female were all associated with increased psychological distress and reduced wellbeing (*p <* 0.05). Psychological distress was more elevated for those with high difficulties adjusting to COVID-19 and high levels of thwarted belongingness (*p <* 0.005). Similarly, as COVID-19-related work and social adjustment difficulties increased, wellbeing reduced. This was more pronounced in those who felt lower levels of loneliness (*p <* 0.0001). Mooney and Becker (2021) monitored undergraduate computing students’ sense of belonging for over 3 years and found that the COVID-19 pandemic had a larger impact on the sense of belonging of all students. They also found that men and women who do not identify as being part of any minority appear to have had similar downward shifts in their sense of belonging. In addition, men who do not identify as being part of any minority saw the largest statistically significant drop in belongingness post-COVID-19. Zhou et al. (2023) examined loneliness and school belonging as predictors of suicide risk in college students in China in a cross-sectional study. In total, 393 college students participated in the study. The results of hierarchical regression analyses that controlled for age and gender indicated that school belonging buffers the negative effects of loneliness on suicidal behaviour and depression. Evidence of a significant loneliness and school belonging interaction as a predictor of both suicidal behaviour and depression was found. Kusci et al. (2023) conducted research with a sample composed of international students studying in universities in Türkiye. Sense of belonging was found to be in a negative relation with social exclusion and loneliness. Moreover, a positive relationship was observed between social exclusion and loneliness. Finally, it was noticed that social exclusion acts as a mediator of the process towards belonging and loneliness and has a significant impact on loneliness.

The notion of belonging is widely explored in academic literature and is associated with a range of connections to places, experiences and communities (Antonsich, 2010; Dost and Mazzoli Smith, 2023; Dost, 2024a; Dost, 2024b). When considering explicitly loneliness, the most fitting point is social belonging—the subjective bonds and relationships individuals develop with others. Belonging and loneliness are two related constructs that encompass personal experiences that are central to being human, indicative of the ubiquity of social need (Cacioppo and Patrick, 2008). Belongingness, the deep feeling of being connected to something greater than oneself, has long been a key issue in psychological and educational discussion (Allan et al., 2021; Baumeister and Leary, 2017; Dost, 2024a). It can be grounded in Maslow’s hierarchy of needs (Maslow, 1968). This system lists belonging as one of the most basic emotional needs, third only to food and shelter and safety. A lack of belongingness can result in striking degrees of loneliness, isolation and alienation, illustrating the importance of this need in total wellbeing (Allen et al., 2021). The university social membership is also important for the cultivation of a student identity. When these group affiliations are disrupted (as during the pandemic), identity confusion or alienation leads to greater loneliness. In the wake of profound disruptions in the university in recent years, it is crucial to capture the ways in which students have accommodated working conditions over time and to understand this in relation to other constraints—initially during and post-pandemic. Its unclear whether belongingness and loneliness associations became stronger more generally after the pandemic was declared (e.g., Killgore et al., 2020; Sutin et al., 2020). Studies have found similar (Barringer et al., 2023; Gopalan et al., 2022; Peng and Roth, 2021; Sibley et al., 2020), higher (Kovacs et al., 2021; MacDonald and Hülür, 2021), and lower (Bartrés-Faz et al., 2021; Kelly et al., 2024; Morán-Soto et al., 2022) levels of correlations between loneliness and belongingness. This study is designed to fill this gap in the literature on the link between students’ self-concept of belonging and loneliness before, during, and after the COVID-19 pandemic. Understanding the adaptations of university life to pandemic-related institution shutdowns and post-pandemic conditions would enable educational institutions to better comprehend the changing nature of student life and develop more effective strategies to address the next set of challenges. To this end, the present study systematically gathered research and publications from before, during and after the pandemic to discuss the changes in students’ experiences of belonging and loneliness between these different epochs. This argument seeks to add richness to our understanding of how students are traversing their social terrain during an era of dramatic shifts.

### 1.1 Types of loneliness

Loneliness is a complex emotional state which manifests as a deep feeling of social isolation (Rokach, 2004). It happens when there is a sizeable discrepancy between a person’s real social ties and the connections they hope for. It is essential to differentiate between social isolation and loneliness, as the former does not uniformly predict experiences of the latter; feelings of loneliness are shaped by enduring personal traits (Leigh-Hunt et al., 2017; Svendsen, 2017) and various external factors, including personality characteristics (such as introversion, shyness, and heightened sensitivity) (Buecker et al., 2020), desires for social interaction, and expectations regarding social connections (Akhter-Khan et al., 2023), as well as significant life changes (e.g., transitions like starting college) (Drake et al., 2016; Shaver et al., 1985), along with physical and mental health issues (such as chronic illnesses, disabilities, or physical restrictions) (Maes et al., 2017). Additionally, interactions through digital platforms (where excessive social media usage can sometimes result in superficial relationships) (Nowland et al., 2018) and cultural standards (where different cultures exhibit diverse expectations about social connectedness, family dynamics, and independence) (Lykes and Kemmelmeier, 2014) also play a role. These elements may elucidate the heterogeneous impact of the pandemic on loneliness. For instance, the overall trajectory of loneliness since the pandemic’s onset remains ambiguous, with some studies indicating stable levels (Mund et al., 2020; Peng and Roth, 2021) while others report increases (Kovacs et al., 2021; Lee et al., 2020) or even declines (Bartrés-Faz et al., 2021). Possible contributing factors to these mixed findings include the duration of restriction measures (Bartrés-Faz et al., 2021), as well as variations in sampling and study methodologies. This is relevant, because variables such as the frequency of social contacts, cohabitation, and size of social network which are considered to contribute to real life being socially isolated do not act as sufficient predictors for that type of condition, as it is usually described from mental health research (Nicholson, 2012; Zavaleta et al., 2017). Although these objective dimensions are all factors that can contribute to feelings of loneliness, the experience of loneliness is largely determined by how people subjectively perceive and evaluate their relationships. This is both regarding the level of satisfaction they get from existing relationships and their experiences of social inclusion. As they delve into the complexities of loneliness, Cacioppo et al. (2015, 2016) discerned three main forms of this state of being, which can be seen as encompassing its various dimensions: intimate or emotional loneliness, relational or social loneliness and collective loneliness. Emotional loneliness derives from absence of intimacy with a partner assuring validation, support, and companionship. Emotional isolation refers to a deeper, more intimate emptiness – it means feelings of being disconnected from a partner on an emotional level that is not easily remedied. The implications of emotional loneliness can be even more hazardous as it is positively correlated with increased distress and homicidality, adversely affecting mental wellbeing (Park et al., 2020; Stickley and Koyanagi, 2016).

Relational loneliness is defined as not the availability of a supportive partner, family, or friends, who all together make the interpersonal space as bonds of worth with one another (Cacioppo et al., 2015, p. 240). In contrast to these cases, collective loneliness describes a more general feeling state of disconnectedness with wider social groups and communities. It emphasises the need to be a member of social groups outside the walls of personal relationships. This type of loneliness is associated with participation in voluntary groups and involvement in broader social networks – feelings of hovering on the fringes of real social intercourse. Indeed, some people in the midst of crowds feel even more lonely due to an enhanced sense of both anonymity and lack of intimacy. Moreover, social loneliness is described as the absence of a needed network of peers or friends, suggesting a more broader level of social involvement. Temporal and spatial dimensions can also be used to draw the outline of loneliness, such as two basic dimensions including state and trait loneliness (Tam and Chan, 2019). State loneliness refers to a temporary emotional experience which result due to situational factors, such as life transitions (e.g., moving away for a job, or the ending of personal relationships) that lead to periods where an individual is removed from their usual social support network. Conversely, trait loneliness is a more enduring characteristic (i.e., persisting even when personal circumstances do not warrant such feelings) characterised by an underlying tendency to experience chronic dissatisfaction in and detachment from social relationships. Knowing the differences between them can help us tailor our approach to different needs that people have when they are coping with feelings of isolation.

### 1.2 Types of belongingness

Humans have an inherent need to establish and maintain lasting and positive relationships with others (Baumeister and Leary, 2017). Rose et al. (2015) note that starting from birth, a person’s wellbeing is closely associated with the nurturing support provided by others. This link is not just about emotional fulfillment; from an evolutionary perspective, a person’s capacity for forming social bonds has had real survival value (Cacioppo and Patrick, 2008; Kenrick and Trost, 2004). People are not only creatures of contact; the nature of belonging is to strive for mutually fulfilling, durable, and supportive relationships (Dost and Mazzoli Smith, 2023). In a comprehensive examination conducted by Dost (2024a), the intricate concept of a general sense of belonging is systematically analysed and categorised into four distinct yet interrelated components: ‘adaptation period sense of belonging’, ‘integration period sense of belonging’, ‘continuum period sense of belonging’, and ‘transition period sense of belonging’. These elements are introduced as part of successive phases within a cumulative cycle, that capture the complexity and progression of belonging. The first component, the “adaptation period sense of belonging,” reflects the starting point of an individual’s journey into a new environment, group, community, or area of study. In this critical phase, participants take on the vital task of making connections and settling into their new surroundings. It is for this reason that this adapting of one utterance by reference to another is vitally important as a basis for subsequent types of interaction and relationship. This is then followed by the ‘integration period sense of belonging’, which is characterised as a more sophisticated and nuanced interactivity among individuals. In this phase, the interactions become more constructive and meaningful, as individuals align their personal values with those resonant within the group or community. It is in this stage that the collaborative spirit flourishes, allowing for the development of social connections that are not only supportive but also conducive to collective learning and growth. The study further elaborates on the ‘continuum period sense of belonging’, which captures the enduring and positive sentiment one maintains toward a particular place, subject matter, or community over an extended period. This stage highlights the importance of maintaining group unity and working toward common goals. It reflects a deep and ongoing expression of support for community, the idea that people feel deeply connected and implicated in relation to their group dynamics. This constant linkage does not only strengthen the individual identity, but also encourages a feeling of responsibility and contribution for the wellbeing of the group. Lastly, the ‘transition period sense of belonging’ emerges as an emotional and psychological experience encountered during intervals between two stable states. This is the critical juncture, it’s one of those transitional phases in our personal journey, prompting necessary preparatory adjustments for what lies ahead. Whether adjusting to new routines, learning new skills or dealing with unexpected circumstances, this period of change is vital both for personal growth and for the development of collective identities.

### 1.3 Study protocol

The current meta-analysis was constructed and executed in accordance with the guidelines of the Preferred Reporting Items for Systematic Review and Meta-Analyses (PRISMA) statement detailed by Moher et al. (2010). This approach promotes clarity and guidance for systematic reviews and meta-analyses, supports rigorous methods throughout all aspects of the research process.

### 1.4 Selection of studies

To ensure a comprehensive review of the literature, a systematic search was undertaken across a range of well-known databases such as Web of Science, Scopus, ERIC, APA PhysicsInfo and the British Education Index. Articles from these digital resources cover a wide variety of academic fields but especially in the areas of social sciences, health, and education. The search was conducted on 12 June 2024. The searches were initially limited to peer reviewed articles published in English with a focus on articles examining belongingness and loneliness across three temporal stages, i.e., before the pandemic, during the pandemic, and post the pandemic. This temporal lens was crucial to understanding the changing politics of these issues in higher education. The reference lists of all articles that met the prespecified inclusion criteria were also manually checked, in addition to the database searches. This was important in order to find any new and relevant studies that might have missed initially and to have a broad and robust pool of data set for analysis.

### 1.5 Inclusion and exclusion criteria

The first criteria set for the inclusion of studies in this study were excellent enough to ensure the relevance and methodological strongness of the selected literature. In general, inclusion criteria stipulated that: (a) participants sampled in the studies needed to be enrolled in accredited institutions of higher education, ensuring the findings applied to a valid academic context, (b) data collection methods needed to be conducted in environments that were directly related to higher education or done through established online platforms that were recognised for scholarly research, (c) the utilisation of a quantitative research methodology was required, which allowed for the extraction of numerical data for statistical analysis, (d) the studies had to specifically evaluate the topic of loneliness and belonging, which are key elements related to student experiences, (e) belonging needed to be treated as a dependent variable, which narrowed the focus to how loneliness is tied to students affiliation, and (f) an effect size had to either be explicitly reported in the studies or be derivable from the analyses reported in peer-reviewed journals, which provided a measure of the strength of the relationship between loneliness and belonging.

Publications meeting the defined inclusion criteria were subjected to further detailed review in stage 2 of the search process, and publications failing to meet these criteria were systematically eliminated. The search did not include any publications that were considered to be unavailable for review. During qualitative assessment, any additional series of exclusion criteria were then implemented to further refine the studies that were included in the pool of studies accepted into the review: (a) studies that were theoretical papers, systematic reviews, meta-analyses, and/or qualitative studies were excluded from this process to keep the focus fully on quantitative studies; (b) any paper that did not directly assess both belongingness and loneliness in higher education students was excluded to maintain relevance; (c) studies that did not have available full text, and/or for which the authors could not provide us with a copy upon our request, were removed from the pool to ensure transparency and rigorous examination of the pool; (d) any paper that did not report or could not provide statistical information on the relationship found between loneliness and belonging were excluded to keep all data included self-derived; and (e) any duplicated papers (e.g., from different databases) and/or book chapters that were the same as a paper that had been retrieved in the journal articles were excluded, as journal articles typically offered more in-depth and comprehensive analysis.

After compilation of the publications following the initial criteria, the researcher used a set of criteria to further refine the studies selected for the subsequent meta-analysis (stage 3). These stringent inclusion criteria were designed to secure the quality of the inclusion and were as follows: (a) only papers that reported at least one aspect of intervention loneliness and belongingness among higher education students with underlying statistical evidence were included; (b) studies that conducted a rigorous assessment of participants’ levels of loneliness and belonging were given higher priority, and studies that provided sufficient quantitative data to calculate effect sizes were included; and (c) studies that discussed the correlation between loneliness and belongingness among higher education students were included. By rigorously applying these more stringent criteria the researcher hoped to build a solid and relevant synthesis to the literature on these important issues to higher education of loneliness and belonging and to offer some new thoughts and reflections to the field.

## 2 Materials and methods

### 2.1 Study selection

**Figure 1:**
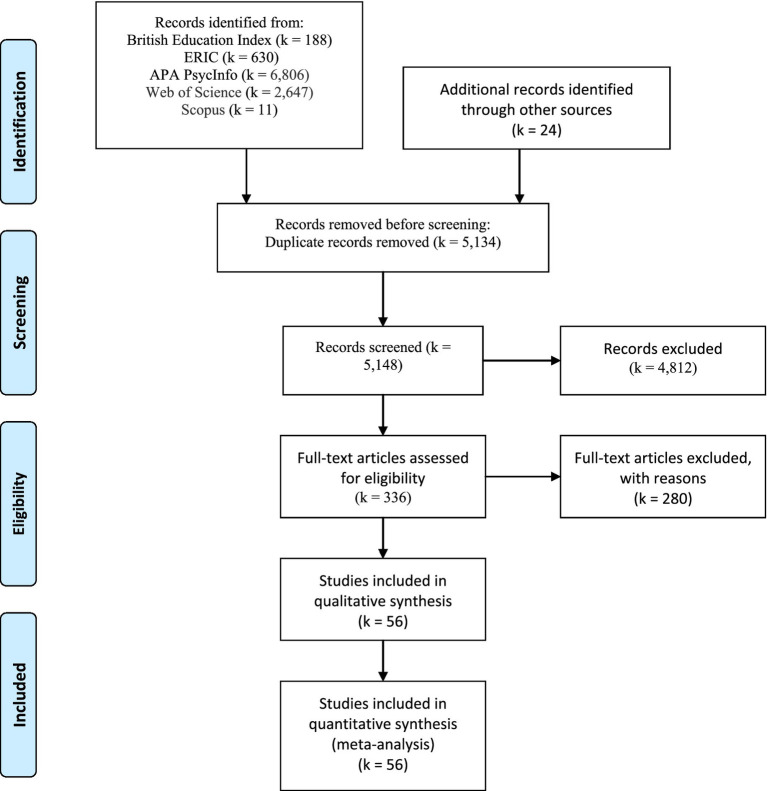
PRISMA flow diagram showing the result of the search and screening process. From: Moher et al. (2010).

Pilot selection procedure was carried out on a random sample of studies before formal screening. Figure 1 demonstrates the flow of study selection according to the Preferred Reporting Items for Systematic and Meta-analyses (PRISMA) guidelines (Moher et al., 2010). An initial pool of 10,282 papers was discovered, which were then screened (see Figure 1). After removing duplicates, all the studies obtained in the search phase were reviewed through their title and abstract using inclusion and exclusion criteria as below. Moreover, after obtaining full-text articles, all articles were hand-searched to identify their eligibility and eligible articles were chosen based on inclusion/exclusion criteria. The researcher first screened studies by sample age and method being in the quantitative form and written in English and from English-speaking countries. Thus, 4,812 articles were excluded due to this first screening. Four, a closer examination of the analysis and statistical results revealed studies in which one or more themes were identified as independent variables and those which identified dependent variables, for which effect sizes were present or could be computed. This resulted in the exclusion of 280 records. A final review resulted in the elimination of studies due to absent data or inadequate measurement, culminating in a final total of 56 studies.

*Search string:* ‘University belong*’ OR ‘college belong*’ OR ‘higher education belong*’ OR ‘tertiary education’ AND ‘belong*’ OR ‘college belong*’ OR ‘undergraduate belong*’ OR ‘University attach*’ OR ‘college attach*‘OR ‘higher education attach*’ OR ‘tertiary education attach*’ OR ‘college attach*’ OR ‘undergraduate attach*’ OR ‘University bond*’ OR ‘college bond*’ OR ‘higher education bond*’ OR ‘tertiary education bond*’ OR ‘college bond*’ OR ‘undergraduate bond*‘OR ‘University connect*’ OR ‘college connect*’ OR ‘higher education connect*’ OR ‘tertiary education connect*’ OR ‘college connect*’ OR ‘undergraduate connect*’ OR ‘University relate*’ OR ‘college relate*‘OR ‘higher education relate*’ OR ‘tertiary education relate*’ OR ‘college relate*’ OR ‘undergraduate relate*‘OR ‘University engag*’ OR ‘college engag*’ OR ‘higher education engag*’ OR ‘tertiary education engag*’ OR ‘college engag*’ OR ‘undergraduate engag*’ AND ‘loneliness’ OR ‘social isolation’ OR ‘social exclusion’ OR ‘lonely or aloneness’ OR ‘solitude’ or ‘lack social interaction’ OR ‘alienation’ OR ‘psychological distance’ OR ‘social deprivation’ OR ‘solitariness’ OR ‘social withdrawal’ OR ‘covid-19’ OR ‘coronavirus’ OR ‘2019-ncov’ OR ‘sars-cov-2′ v ‘cov-19’ OR ‘2019 pandemic’ OR ‘pandemic’ OR ‘lockdown’ AND ‘belongingness’ OR ‘connectedness’ OR ‘belonging’ OR ‘community’ OR ‘sense of community’ OR ‘sense of belongingness’ OR ‘belong*’ AND ‘higher education’, OR ‘university’, OR ‘undergraduate’, OR ‘undergraduate students’, OR ‘university students’.

### 2.2 Data extraction and coding

The author categorised all studies in a comprehensive spread sheet. The following information was extracted from each study: year of the study, location (i.e., the country from which data was collected), the sample size, the range of participants’ age, mean scores for belonging, mean scores for loneliness; standard deviations (SD) for belonging and loneliness scores, the length of follow-up, if any; and the measures used to assess loneliness and belonging as well as the mean and standard deviation calculated on the sample; and bivariate correlation (Pearson’s r) or alternative effect size measures of the relationship between belonging and loneliness.

### 2.3 Quality assessment

The quality of full-text articles was critically appraised using the Sieve Quality Assessment Criteria, a unique methodology of appraisal created by Gorard et al. (2020) (see Table 1). The Sieve criteria highlight the complexities involved in research and analytic designs (e.g., education and the social sciences). The methodological quality and the topical relevance of the studies were assessed in detail by the researcher in order to identify the best quality studies. The evaluation process was systematic and multidimensional, considering every paper on the basis of a number of well-specified dimensions. With respect to the diligence of the review, the researcher considered the precision of the research questions, the adequacy of the methods that were used, the trustworthiness and validity of the data collection methods, and the transparency of the data analysis. In order to filter articles not meeting pre-specified quality criterions but including only articles presented through rigorous and transparent research methodologies the study aspired at the use of these specific indications. Each study considered in this analysis was also subjected to a second review of the security of its evidence, based on what Gorard (2024) calls the ‘sieve’ and reflecting consideration of five key criteria: (a) the research design and its fit with the study question, for example, whether the study is an Randomized Controlled Trial (RCT) with random assignment, a matched comparison, a longitudinal cohort study; (b) the scale of the study, in particular the smaller size in any substantive comparison; (c) the level of attrition or study drop out; (d) the quality of the outcome measurement, whether it was based on self-reports, use of administrative data, standardised assessments, or evaluations related to the intervention; and (e) other potential threats to validity, such as compromised randomisation or conflicts of interest.

**Table 1:** Quality appraisal ‘sieve’ (for causal studies) adapted from Gorard et al. (2020).

Design	Scale	Dropout	Outcomes	Other threats	Rating
Fair design for comparison (e.g., RCT)	Large number of cases per comparison group	Minimal attrition with no evidence that it affects the outcomes	Standardised pre-specified independent outcome	No evidence of diffusion or other threat	4*
Balanced comparison (e.g., Regression Discontinuity, Difference-in Difference)	Medium number of cases per comparison group	Some initial imbalance or attrition	Pre-specified outcome, not standardised or not independent	Indication of diffusion or other threat, unintended variation in delivery	3*
Matched comparison (e.g., propensity score matching)	Small number of cases per comparison group	Initial imbalance or moderate attrition	Not pre-specified, but valid outcome	Evidence of experimenter effect, diffusion or variation in delivery	2*
Comparison with poor or no equivalence (e.g., comparing volunteers with non-volunteers)	Very small number of cases pr comparison group	Substantial imbalance or high attrition	Outcomes with issues of validity and appropriateness	Strong indication of diffusion or poorly specified	1*
No report of comparator	A trivial scale of study (or N unclear)	Attrition not reported or too high for comparison	Too many outcomes, weak measures or poor reliability	No consideration of threats to validity	0

This rigorous process of evaluation was fundamental to guaranteeing that the results and recommendations of the research were based firmly on the quality and reliability of the evidence available. In order to have a good evaluation of evidence base, this study developed a scoring system based on stringent criteria. Each study was scored by one of the researcher using a modified scoring system ranging from 0 to 4 for topic relevance. A score of 0 indicates that this study is not qualified to be included in this meta analysis and 1* is the minimal score for a study for inclusion in the review. A score of 4* indicates the strongest degree of causal evidence achievable from real world studies (high reliability and validity) while 0 identifies the weakest. This score serves as a tool for assessing the safety and reliability of the evidence derived from the studies included. The rating system helps determine which studies are the best suited for making causal claims about the phenomenon(s) in question. As Gorard et al. (2017) have stressed, research designs vary in their degree of rigour, and are often organised in a hierarchy. For example, randomised controlled trials (RCTs), which are widely considered the most rigorous study design because they have the greatest potential to minimise bias, are given the highest rating of 4*. The studies which utilise convenient comparison groups are scored as 1*. In contrast, quasi-experimental designs, which are intermediate between the other two types of designs, mostly fall between 2* and 3*, indicating moderate rigour and trustworthiness. The results showed that 21 studies were scored 2* of a maximum of four, which suggested moderate quality and relevance of those studies examined (see Table 2). In addition, 24 studies scored 3* indicating of greater importance or rigour. Eleven studies met the highest quality and rigour criteria (score 4*). Such systematic analysis helped in over categorisation of the studies and depicting the overall contribution and impact of these studies on the field.

**Table 2:** Quality appraisal ‘sieve’ for included studies.

Author/s	Year	Design	Scale	Dropout	Outcomes	Other threats	Rating
Alkan	2014	Balanced comparison (e.g., regression discontinuity, difference-in difference)	Medium number of cases per comparison group	Some initial imbalance or attrition	Pre-specified outcome, not standardised or not independent	Indication of diffusion or other threat, unintended variation in delivery	3*
Arslan	2021	Balanced comparison (e.g., regression discontinuity, difference-in difference)	Medium number of cases per comparison group	Some initial imbalance or attrition	Pre-specified outcome, not standardised or not independent	Indication of diffusion or other threat, unintended variation in delivery	3*
Thomas, Orme, and Kerrigan	2020	Balanced comparison (e.g., regression discontinuity, difference-in difference)	Medium number of cases per comparison group	Some initial imbalance or attrition	Pre-specified outcome, not standardised or not independent	Indication of diffusion or other threat, unintended variation in delivery	3*
Wilson and Liss	2023	Matched comparison (e.g., propensity score matching)	Small number of cases per comparison group	Initial imbalance or moderate attrition	Not pre-specified, but valid outcome	Evidence of experimenter effect, diffusion or variation in delivery	2*
Dingle, Han, and Carlyle	2022	Fair design for comparison (e.g., randomised controlled trial)	Large number of cases per comparison group	Minimal attrition with no evidence that it affects the outcomes	Standardised pre-specified independent outcome	No evidence of diffusion or other threat	4*
Dopmeijer et al.	2022	Fair design for comparison (e.g., randomised controlled trial)	Large number of cases per comparison group	Minimal attrition with no evidence that it affects the outcomes	Standardised pre-specified independent outcome	No evidence of diffusion or other threat	4*
Zhou et al.	2023	Balanced comparison (e.g., regression discontinuity, difference-in difference)	Medium number of cases per comparison group	Some initial imbalance or attrition	Pre-specified outcome, not standardised or not independent	Indication of diffusion or other threat, unintended variation in delivery	3*
Satan	2020	Matched comparison (e.g., propensity score matching)	Small number of cases per comparison group	Initial imbalance or moderate attrition	Not pre-specified, but valid outcome	Evidence of experimenter effect, diffusion or variation in delivery	2*
Lange and Crawford	2024	Balanced comparison (e.g., regression discontinuity, difference-in difference)	Medium number of cases per comparison group	Some initial imbalance or attrition	Pre-specified outcome, not standardised or not independent	Indication of diffusion or other threat, unintended variation in delivery	3*
Kennedy and Tuckman	2013	Balanced comparison (e.g., regression discontinuity, difference-in difference)	Medium number of cases per comparison group	Some initial imbalance or attrition	Pre-specified outcome, not standardised or not independent	Indication of diffusion or other threat, unintended variation in delivery	3*
Wei et al.	2005	Matched comparison (e.g., propensity score matching)	Small number of cases per comparison group	Initial imbalance or moderate attrition	Not pre-specified, but valid outcome	Evidence of experimenter effect, diffusion or variation in delivery	2*
Yoo, Park and Jun	2014	Matched comparison (e.g., propensity score matching)	Small number of cases per comparison group	Initial imbalance or moderate attrition	Not pre-specified, but valid outcome	Evidence of experimenter effect, diffusion or variation in delivery	2*
Alkan	2016	Balanced comparison (e.g., regression discontinuity, difference-in difference)	Medium number of cases per comparison group	Some initial imbalance or attrition	Pre-specified outcome, not standardised or not independent	Indication of diffusion or other threat, unintended variation in delivery	3*
Chen and Chung	2007	Matched comparison (e.g., propensity score matching)	Small number of cases per comparison group	Initial imbalance or moderate attrition	Not pre-specified, but valid outcome	Evidence of experimenter effect, diffusion or variation in delivery	2*
Satici, Uysal, and Deniz	2016	Matched comparison (e.g., propensity score matching)	Small number of cases per comparison group	Initial imbalance or moderate attrition	Not pre-specified, but valid outcome	Evidence of experimenter effect, diffusion or variation in delivery	2*
Hopmeyer and Medovoy	2017	Balanced comparison (e.g., regression discontinuity, difference-in difference)	Medium number of cases per comparison group	Some initial imbalance or attrition	Pre-specified outcome, not standardised or not independent	Indication of diffusion or other threat, unintended variation in delivery	3*
Turton et al.	2018	Matched comparison (e.g., propensity score matching)	Small number of cases per comparison group	Initial imbalance or moderate attrition	Not pre-specified, but valid outcome	Evidence of experimenter effect, diffusion or variation in delivery	2*
Stice and Lavner	2019	Fair design for comparison (e.g., randomised controlled trial)	Large number of cases per comparison group	Minimal attrition with no evidence that it affects the outcomes	Standardised pre-specified independent outcome	No evidence of diffusion or other threat	4*
Seo	2020	Matched comparison (e.g., propensity score matching)	Small number of cases per comparison group	Initial imbalance or moderate attrition	Not pre-specified, but valid outcome	Evidence of experimenter effect, diffusion or variation in delivery	2*
Wax et al.	2019	Fair design for comparison (e.g., randomised controlled trial)	Large number of cases per comparison group	Minimal attrition with no evidence that it affects the outcomes	Standardised pre-specified independent outcome	No evidence of diffusion or other threat	4*
Nguyen, Werner, and Soenens	2019	Matched comparison (e.g., propensity score matching)	Small number of cases per comparison group	Initial imbalance or moderate attrition	Not pre-specified, but valid outcome	Evidence of experimenter effect, diffusion or variation in delivery	2*
Ko et al.	2022	Balanced comparison (e.g., regression discontinuity, difference-in difference)	Medium number of cases per comparison group	Some initial imbalance or attrition	Pre-specified outcome, not standardised or not independent	Indication of diffusion or other threat, unintended variation in delivery	3*
Datu and Fincham	2022	Fair design for comparison (e.g., randomised controlled trial)	Large number of cases per comparison group	Minimal attrition with no evidence that it affects the outcomes	Standardised pre-specified independent outcome	No evidence of diffusion or other threat	4*
Brunsting et al.	2021	Comparison with poor or no equivalence (e.g., comparing volunteers with non-volunteers)	Very small number of cases pr comparison group	Substantial imbalance or high attrition	Outcomes with issues of validity and appropriateness	Strong indication of diffusion or poorly specified approach	1*
Low, Bhar & Chen	2023	Comparison with poor or no equivalence (e.g., comparing volunteers with non-volunteers)	Very small number of cases pr comparison group	Substantial imbalance or high attrition	Outcomes with issues of validity and appropriateness	Strong indication of diffusion or poorly specified approach	1*
Abebe et al.	2024	Fair design for comparison (e.g., randomised controlled trial)	Large number of cases per comparison group	Minimal attrition with no evidence that it affects the outcomes	Standardised pre-specified independent outcome	No evidence of diffusion or other threat	4*
Zou and Mu	2024	Balanced comparison (e.g., regression discontinuity, difference-in difference)	Medium number of cases per comparison group	Some initial imbalance or attrition	Pre-specified outcome, not standardised or not independent	Indication of diffusion or other threat, unintended variation in delivery	3*
Beloborodova et al.	2024	Balanced comparison (e.g., regression discontinuity, difference-in difference)	Medium number of cases per comparison group	Some initial imbalance or attrition	Pre-specified outcome, not standardised or not independent	Indication of diffusion or other threat, unintended variation in delivery	3*
Kusci, Oztosun, and Arli	2023	Comparison with poor or no equivalence (e.g., comparing volunteers with non-volunteers)	Very small number of cases pr comparison group	Substantial imbalance or high attrition	Outcomes with issues of validity and appropriateness	Strong indication of diffusion or poorly specified approach	1*
Kochel, Bagwell and Abrash	2022	Balanced comparison (e.g., regression discontinuity, difference-in difference)	Medium number of cases per comparison group	Some initial imbalance or attrition	Pre-specified outcome, not standardised or not independent	Indication of diffusion or other threat, unintended variation in delivery	3*
Ouzia, Wong and Dommett	2023	Matched comparison (e.g., propensity score matching)	Small number of cases per comparison group	Initial imbalance or moderate attrition	Not pre-specified, but valid outcome	Evidence of experimenter effect, diffusion or variation in delivery	2*
Mellinger et al.	2024	Matched comparison (e.g., propensity score matching)	Small number of cases per comparison group	Initial imbalance or moderate attrition	Not pre-specified, but valid outcome	Evidence of experimenter effect, diffusion or variation in delivery	2*
Morse et al.	2021	Matched comparison (e.g., propensity score matching)	Small number of cases per comparison group	Initial imbalance or moderate attrition	Not pre-specified, but valid outcome	Evidence of experimenter effect, diffusion or variation in delivery	2*
Karababa and Tayli	2020	Balanced comparison (e.g., regression discontinuity, difference-in difference)	Medium number of cases per comparison group	Some initial imbalance or attrition	Pre-specified outcome, not standardised or not independent	Indication of diffusion or other threat, unintended variation in delivery	3*
Blankenau et al.	2023	Balanced comparison (e.g., regression discontinuity, difference-in difference)	Medium number of cases per comparison group	Some initial imbalance or attrition	Pre-specified outcome, not standardised or not independent	Indication of diffusion or other threat, unintended variation in delivery	3*
Worsley, Harrison, and Corcoran	2023	Fair design for comparison (e.g., randomised controlled trial)	Large number of cases per comparison group	Minimal attrition with no evidence that it affects the outcomes	Standardised pre- specified independent outcome	No evidence of diffusion or other threat	4*
Knifsend	2020	Matched comparison (e.g., propensity score matching)	Small number of cases per comparison group	Initial imbalance or moderate attrition	Not pre-specified, but valid outcome	Evidence of experimenter effect, diffusion or variation in delivery	2*
Andreadis et al.	2023	Matched comparison (e.g., propensity score matching)	Small number of cases per comparison group	Initial imbalance or moderate attrition	Not pre-specified, but valid outcome	Evidence of experimenter effect, diffusion or variation in delivery	2*
Mounts	2004	Matched comparison (e.g., propensity score matching)	Small number of cases per comparison group	Initial imbalance or moderate attrition	Not pre-specified, but valid outcome	Evidence of experimenter effect, diffusion or variation in delivery	2*
Hansen-Brown et al.	2022	Matched comparison (e.g., propensity score matching)	Small number of cases per comparison group	Initial imbalance or moderate attrition	Not pre-specified, but valid outcome	Evidence of experimenter effect, diffusion or variation in delivery	2*
Brance et al.	2022	Comparison with poor or no equivalence (e.g., comparing volunteers with non-volunteers)	Very small number of cases pr comparison group	Substantial imbalance or high attrition	Outcomes with issues of validity and appropriateness	Strong indication of diffusion or poorly specified approach	1*
Arslan, Yildirim, Zangeneh	2021	Comparison with poor or no equivalence (e.g., comparing volunteers with non-volunteers)	Very small number of cases pr comparison group	Substantial imbalance or high attrition	Outcomes with issues of validity and appropriateness	Strong indication of diffusion or poorly specified approach	1*
Hohne et al.	2022	Balanced comparison (e.g., regression discontinuity, difference-in difference)	Medium number of cases per comparison group	Some initial imbalance or attrition	Pre-specified outcome, not standardised or not independent	Indication of diffusion or other threat, unintended variation in delivery	3*
Sebekova and Uhlarikova	2023	Comparison with poor or no equivalence (e.g., comparing volunteers with non-volunteers)	Very small number of cases pr comparison group	Substantial imbalance or high attrition	Outcomes with issues of validity and appropriateness	Strong indication of diffusion or poorly specified approach	1*
McFayden et al.	2023	Balanced comparison (e.g., regression discontinuity, difference-in difference)	Medium number of cases per comparison group	Some initial imbalance or attrition	Pre-specified outcome, not standardised or not independent	Indication of diffusion or other threat, unintended variation in delivery	3*
Werner et al.	2021	Balanced comparison (e.g., regression discontinuity, difference-in difference)	Medium number of cases per comparison group	Some initial imbalance or attrition	Pre-specified outcome, not standardised or not independent	Indication of diffusion or other threat, unintended variation in delivery	3*
Mäkiniemi, Oksanen, & Mäkikangas	2021	Fair design for comparison (e.g., randomised controlled trial)	Large number of cases per comparison group	Minimal attrition with no evidence that it affects the outcomes	Standardised pre- specified independent outcome	No evidence of diffusion or other threat	4*
Marler et al.	2021	Matched comparison (e.g., propensity score matching)	Small number of cases per comparison group	Initial imbalance or moderate attrition	Not pre-specified, but valid outcome	Evidence of experimenter effect, diffusion or variation in delivery	2*
Padmanabhanunni and Pretorius	2021	Matched comparison (e.g., propensity score matching)	Small number of cases per comparison group	Initial imbalance or moderate attrition	Not pre-specified, but valid outcome	Evidence of experimenter effect, diffusion or variation in delivery	2*
Allan et al.	2021	Balanced comparison (e.g., regression discontinuity, difference-in difference)	Medium number of cases per comparison group	Some initial imbalance or attrition	Pre-specified outcome, not standardised or not independent	Indication of diffusion or other threat, unintended variation in delivery	3*
Graf and Bolling,	2024	Comparison with poor or no equivalence (e.g., comparing volunteers with non-volunteers)	Very small number of cases pr comparison group	Substantial imbalance or high attrition	Outcomes with issues of validity and appropriateness	Strong indication of diffusion or poorly specified approach	1*
Berman et al.	2022	Fair design for comparison (e.g., randomised controlled trial)	Large number of cases per comparison group	Minimal attrition with no evidence that it affects the outcomes	Standardised pre- specified independent outcome	No evidence of diffusion or other threat	4*
Bonsaksen et al.	2022	Balanced comparison (e.g., regression discontinuity, difference-in difference)	Medium number of cases per comparison group	Some initial imbalance or attrition	Pre-specified outcome, not standardised or not independent	Indication of diffusion or other threat, unintended variation in delivery	3*
Weber et al.	2022	Balanced comparison (e.g., regression discontinuity, difference-in difference)	Medium number of cases per comparison group	Some initial imbalance or attrition	Pre-specified outcome, not standardised or not independent	Indication of diffusion or other threat, unintended variation in delivery	3*
Akkaya and Duy	2024	Balanced comparison (e.g., regression discontinuity, difference-in difference)	Medium number of cases per comparison group	Some initial imbalance or attrition	Pre-specified outcome, not standardised or not independent	Indication of diffusion or other threat, unintended variation in delivery	3*
Samadieh and Rezaei	2024	Balanced comparison (e.g., regression discontinuity, difference-in difference)	Medium number of cases per comparison group	Some initial imbalance or attrition	Pre-specified outcome, not standardised or not independent	Indication of diffusion or other threat, unintended variation in delivery	3*

### 2.4 Effect size extraction

The nature of university students’ loneliness and belonging complexes was explored through an exploratory approach using the correlation coefficient (Pearson r) as the primary survey method. This facilitated an in-depth assessment of the nature and strength of the investigated relationships. The methods employed were *N =* 54 and varied, but more attention could have been focused on those examining effect sizes (important because these measure not only the relationship but also its direction/consistency across studies) (Deeks et al., 2019). While reading the literature, the author encountered a number of methods used to calculate an effect size, followed by another method (such as an alternative statistical method, such as the F test). Comparisons were made across studies, and due to consistency considerations, the author converted the different effect sizes to r to keep the analysis as homogeneous as possible. For example, the author calculated Pearson’s r correlation coefficients and the separate Packard correlation from the reported means and standard deviations using the Lyons Morris calculator at: www.lyonsmorris.com/ma1/index.cfm, a well-known resource. In contrast, he used the Campbell Collaboration calculator at: www.campbellcollaboration.org/escalc/html/EffectSizeCalculator-R6.php to calculate the associated effect sizes. By using both methods, the researcher was able to refine the analysis and increase confidence in the findings because it allowed us to rely more rigorously on the different study designs.

The researcher followed a detailed protocol to avoid confounding effect sizes that went on to the subsequent phase of analysis. This protocol specified that only one effect size could be derived for any unique sample from a given study. Where studies produced more than one effect size or for the same sample on the exact same analysis, the researcher applied carefully crafted strategic rules to control for any potential dependencies. For example, weight was given to longitudinal effects when both cross-sectional and longitudinal relations were provided, because longitudinal findings provide a more informed picture by observing changes over time. Furthermore, in cases where cross-sectional data were reported at multiple time points but no matched longitudinal analyses were available, pooling of the coefficients was done in a holistic approach in order to obtain a single aggregated effect size. The purpose of this approach was to provide a more complete and relevant representation of the data set. In cases where several independent measurement instruments for the same construct were used (e.g., different questionnaires for anxiety), these measures were pooled to a single effect size. This concatenation also served to decrease redundancy and retain data integrity. When there were more than one longitudinal effect size across two constructs with time lags, then the researcher cumulated across the effect sizes. This approach produced an effect size global in time so that a more general relationship over time was modeled, better capturing the temporal dynamics of the data. The researcher used Cohen (1992) guidelines for interpreting effect sizes, suggesting that values of 0.20, 0.50, and 0.80 for Cohen’s d (or Hedges’ g) represent small, medium, and large effects, respectively; while for Pearson’s r, these values were 0.10, 0.30, and 0.50. In order to accommodate for the diversity in sample characteristics and measurement approaches regarding belonging and loneliness, and average effect size was calculated using a random effects model. This model was particularly appropriate because it assumes that the effect size obtained in the studies refers to different populations and not to the same (homogeneous) population. This yielded that the true effect size from the collected studies was a random variable that contained not only the true effect sizes, but also the variances due to sampling error as well as inter-study variation. Once the true effect size estimate had been computed by the researcher using the random-effects model, the overall Fisher’s z-score was further transformed back into Pearson’s r as the correlation coefficient. This conversion was more than a technical conversion; it greatly improved the interpretation of the data and facilitated communication of the results. Clarity of reporting is critical in particular when considering the subtle interrelations between feelings of loneliness and feelings of belonging among higher education students.

### 2.5 Data analysis

This research employed random effects models to succinctly capture the variability in true effect sizes across multiple studies. Variation can in turn be attributed to heterogeneity in country-specific populations or to divergent sampling procedures or research designs (Erez et al., 1996). By using this analytical approach, they expected to obtain a more conservative and stable estimate of an average effect, as expressed by Hedges and Vevea (1998). Given the non-independence of effect size estimates within studies and to reduce the risk of model misspecification, the multi-level modelling method was employed along with a robust variance estimator (RVE) (Pustejovsky and Tipton, 2022; Vite et al., 2024). This methodological approach allowed to include more detailed information in an analysis that does not ignore that effect sizes usually do not occur independently and that shared study characteristics can drive their magnitudes. The heterogeneity of effect sizes was examined well using the statistical methods: Q statistics, tau-squared (τ^2^) and I-squared (I^2^) (Kabir et al., 2024).

A large Q statistic suggests that the variance in effect sizes is not due to sampling error alone and indicates a need to further examine the reasons behind the heterogeneity (Mikolajewicz and Komarova, 2019). The examination of τ^2^ and I^2^ was important to judge how much of the variance could be attributed to between-studies as opposed to within-study sampling error (Lin, 2018). In addition, 95% CIs based on the weighted average effects were calculated following Borenstein et al. (2021). For these confidence intervals, the researcher used cluster robust variance estimation (CRVE) and used small sample degrees of freedom corrections with critical values (Pustejovsky and Tipton, 2018). The factors associated with the variation of correlations between loneliness and belongingness in higher education students. The study additionally investigated the moderators of variance in the correlation between loneliness and belongingness among higher education students through mixed-effects meta-regression models. This study in particular focused on the effect of variables such as publication status, study region, school level, and mean age. Finally, the study evaluated publication bias using a funnel plot asymmetry test, with effect size dependence considered through Egger’s regression test. This meta-analysis not only revealed the evidence of publication bias, but also tested the publication status as a moderator by way of conducting the meta-regression models, which borrowed methodologies from the method developed by Egger et al. (1997) and Rodgers and Pustejovsky (2021). The broad scope of this model made it possible to gain an overview of the factors that might underlie the associations of loneliness and belongingness reported in higher education literature.

*Heterogeneity of effect sizes*: To test if there was significant unexplained heterogeneity between study effects, the researcher sought to ascertain if the variance in effects across samples was larger than the variance that would be expected in a single sample. Such assessment is important since it guides whether it is reasonable to examine moderators of unexplained heterogeneity that might help understanding the origins of such heterogeneity (Cooper et al., 2009). The analysis was conducted by the researcher was the Q-statistic, a principal statistical measure that examines the null hypothesis. This null hypothesis states that the effect sizes estimated in the different studies are sampled from a single, homogeneous population (Nakagawa and Cuthill, 2007). Conversely, the alternative hypothesis states that the variation in effect sizes is larger than would be expected due to sampling error alone, and that the studies might be sampling different population parameters (Kelley and Preacher, 2012; Cochran, 1954). The researcher also conducted I^2^ test, in addition to the Q-statistic. This among-study variability metric informs us about the degree of variation across the studies in how the treatment works and helps us to determine how much of that variation can be real (non-artificial) heterogeneity rather than just sampling error (Huedo-Medina et al., 2006; von Hippel, 2015). I^2^ is the percentage of total variation across studies that is due to heterogeneity and not to chance (Higgins and Thompson, 2002). I^2^ values of 25% are indicative for low heterogeneity between-study, values of 50% for moderate heterogeneity, and values of 75% for high heterogeneity (Higgins and Thompson, 2002; Melsen et al., 2014). These statistical analyses are important to know the reliability of the combined ES and to check if other factors may be confounding the association between loneliness/social isolation and belongingness. These two statistics allow a comprehensive assessment of the consistency and replicability of the results within studies, serving as the basis for further analyses of potential moderating variables (Sanchez et al., 2024).

**Figure 2:**
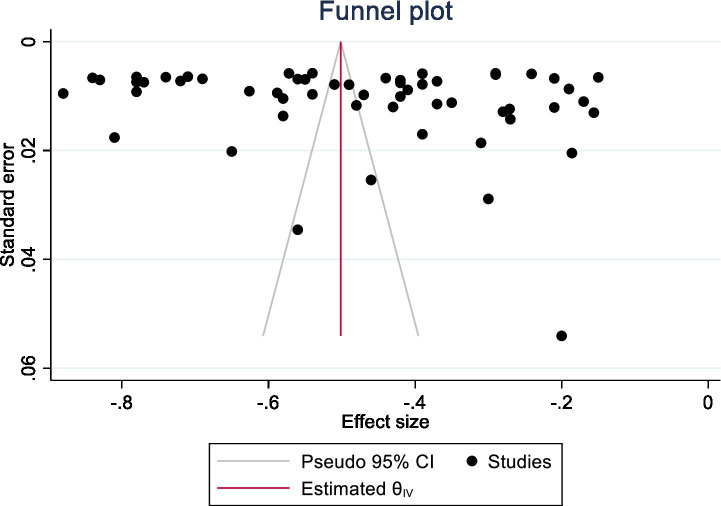
Funnel plot of meta-analysis of published studies. Each plotted point represents the standard error and effect size difference between loneliness and belongingness among higher education students before and during/after the pandemic. The white triangle represents the region where 95% of the data points would lie in the absence of a publication bias. The vertical line represents the average effect size difference of −0.48 found in the meta-analysis.

*Publication bias*: The publication bias was thoroughly investigated using a battery of indices on a wide array of studies by the researcher. The first part of this step-by-step approach was a comparative analysis that sought to compare effect sizes from published studies against those coming from unpublished ones. The analysis sought to detect major differences in correlation findings (and thus the potential effect of publication status on findings in the literature). After this initial examination, the researcher, then, examined the spread of effect sizes within the meta-analytic sample. The researcher sought to identify whether these effect sizes were evenly distributed on both sides of the overall average, making a condition for a balanced representation of the results. On the other hand, an asymmetrical pattern of distribution may suggest bias, which in turn reflects a distorted presentation of the results (Patton, 2004). The comprehensive strategy combined the visual and statistical approach, with particular emphasis on discrepancies between the effect sizes from larger versus smaller-sample studies (Edmonds and Kennedy, 2016; Morris and DeShon, 2002). Finally, it is important to emphasise that analyses with smaller numbers of studies are often subject to greater publication bias (Begg and Berlin, 1988). These smaller studies are often published only if they find a statistically significant result, while studies with larger samples are more likely to be published regardless of whether their results were positive or negative (O’Mara-Eves et al., 2015). A funnel plot was generated by the researcher (a plot of each effect size against its standard error) (see Figure 2). Plots are weighted using number of individual studies with different sample sizes. In a perfect funnel plot one would ideally observe a symmetrical spread of effect sizes centered around the true population effect size (Schwarzer et al., 2015). As power increases, these effect sizes should be narrower and the plot more precise appearing (Ellis, 2010). If the funnel plot introduces evidence of possible publication bias, this could imply the negative findings (those reflected by either weak or even positive relationship scores with loneliness to belonging) are more likely to occur in conjunction with relatively large standard errors, and consequently cluster in a plot’s lower half areas (Song et al., 2002; Tang and Liu, 2000). ‘Egger test’ was performed to further scrutinise the statistical intricacies of publication bias (Figure 2). The present meta-regression analysis was more sophisticated and used the precision of the effect sizes (i.e., the standard errors) as an important predictor of the correlation coefficients in a multilevel modelling context (Egger et al., 1997; Hagger, 2022; Nakagawa et al., 2023). Such a summary result of this meta-regression would be that the intercept of the dependent variable (the correlation coefficient) was significantly different from zero. Such a discovery would mean that the effect size distribution was not symmetrically distributed about the true population effect size, adding weight to concerns about bias in the published literature. Finally, the researcher used the trim-and-fill procedure with the effect sizes in the meta-analytic sample. This advanced method was implemented to improve the estimated average effect size, to determine if it was statistically significant, and to compensate for any apparent funnel plot asymmetry and exclusion of influential outliers (Duval and Tweedie, 2000). Through the use of this multi-method analysis, the researcher aims to develop a more complex and complete picture of publication bias in the literature on the effect sizes for loneliness and belonging.

### 2.6 Statistical analysis

## 3 Results

### 3.1 Overall study characteristics

A total of 61 studies were included in the analysis. Information about the studies and their effect sizes is summarised in Table 3. The studies were published between 2004 and 2024, with sample sizes ranging from 20 to 2,782 participants, resulting in a total sample size of 30,062. Research was conducted in 18 countries: Australia (k = 3, *N =* 1,397), Canada (k = 2, *N =* 576), China (k = 2, *N =* 904), Finland (k = 1, *N =* 1,463), Germany (k = 3, *N =* 1,302), Greece (k = 2, *N =* 385), Norway (k = 1, *N =* 61), USA (k = 26, *N =* 13,779), UK (k = 4, *N =* 1,750), Philippines (k = 1, *N =* 504), Slovakia (k = 1, *N =* 169), South Africa (k = 1, *N =* 337), South Korea (k = 2, *N =* 605), Turkey (k = 9, *N =* 3,312), Taiwan (k = 1, *N =* 319), Iran (k = 1, *N =* 345), and the Netherlands (k = 1, *N =* 3,134). All studies documented a statistically significant negative association between loneliness and belongingness. Individual effect sizes ranged from r = −0.15 to r = −0.83, with most effects classified as moderate in size.

**Table 3:** Study characteristics.

Group	Author/s	Year	Country/ies	N	M (age)	Scale	Types of loneliness	Types of belonging	M (belonging)	SD (belonging)	M (Loneliness)	SD (loneliness)	r (Correlational coefficient)
A	Alkan	2014	Turkey	164	21.67	Psychological Sense of School Membership (PSSM) Scale (Goodenow, 1993) and UCLA Loneliness Scale (Russell et al., 1980; Russell, 1996)	Emotional Loneliness and Subjective Loneliness	Sense of School Membership	3.26	0.49	2.04	0.59	−0.29
B	Arslan	2021	Turkey	333	21.94	College Belonging Scale (Arslan and Duru, 2017) and UCLA Loneliness Scale (ULS-8) (Russell et al., 1978)	Emotional Loneliness and Subjective Loneliness	College Belonging	46.64	9.09	14.51	4.68	−0.54
A	Thomas, Orme, and Kerrigan	2020	UK	510	na	Three-Factor Psychological Sense of Community scale (PSC) (Jason et al., 2015) and The 20-item revised UCLA Loneliness scale (Russell et al., 1980)	Emotional Loneliness and Subjective Loneliness	Psychological sense of community (Membership, Influence, and Needs Fulfillment)	3.9	0.6	2.4	0.7	−0.572
A	Wilson and Liss	2023	USA	372	20.61	The Wake Forest University Well Being Assessment (Brocato et al., 2020; Brocato & Jayawickreme, 2017) (three items sense of belonging subscale, and four items Loneliness subscale)	Subjective, social, and emotional loneliness	Social Belonging	48.04	10.67	53.06	11.45	−0.39
B	Dingle, Han, and Carlyle	2022	Australia	1,239	20.7	The three items UCLA Loneliness Scale (Hughes et al., 2004) and the single-item social identity scale (Postmes et al., 2013).	Emotional Loneliness and Subjective Loneliness	Social identification (Self-definition and Group attachment)	3.39	0.93	4.77	1.63	−0.241
A	Dopmeijer et al.	2022	the Netherlands	3,134	21.8	The De Jong-Gierveld Loneliness Scale (De Jong-Gierveld and van Tilburg, 2006) and the Sense of Belonging questionnaire (Meeuwisse et al., 2010)	Emotional loneliness and social loneliness	Academic Belonging (Institutional Belonging) and Social Belonging (Peer Belonging)	3.81	0.6	na	na	−0.29
A	Zhou et al.	2023	China	393	na	The Chinese version of the Psychological Sense of School Membership (C-PSSM; Cheung and Hui, 2003) and the UCLA Loneliness Scale (Russell, 1996).	Emotional Loneliness and Subjective Loneliness	Psychological Sense of School Membership	75.37	12.71	42.72	10.52	−0.71
A	Satan	2020	Turkey	271	na	UCLA Loneliness Scale (Russel et al., 1980) and Social Connectedness Scale (Sarıçam and Deveci, 2017)	Emotional Loneliness and Subjective Loneliness	Social Connectedness (subjective sense of interpersonal closeness and connection with other)	na	na	na	na	−0.78
B	Lange and Crawford	2024	USA	280	23.61	The 8-item Social Connectedness Scale (SCS; Lee and Robbins, 1995) and the UCLA Loneliness Scale is a 20-item scale (Russell, 1996)	Emotional Loneliness and Subjective Loneliness	subjective sense of interpersonal closeness and connectedness with others and society	na	na	na	na	−0.74
A	Kennedy and Tuckman	2013	USA	671	18.2	Social Exclusion Concerns (CSE) (Kennedy and Tuckman, 2013) and the Psychological Sense of School Membership (PSSM) Scale (Goodenow, 1993)	anticipatory distress and hyperawareness of exclusion	Social belonging and Sense of School Membership	na	na	na	na	−0.15
A	Wei et al.	2005	USA	299	19.73	Basic psychological needs satisfaction (Ilardi et al., 1993) and the UCLA scale (Russell, 1996)	Emotional Loneliness and Subjective Loneliness	Relational Belonging	na	na	na	na	−0.84
A	Yoo, Park and Jun	2014	Korea	304	21.53	Peer Connectedness Scale developed for the Korea Youth Panel Survey VI (National Youth Policy Institute, 2008) and social isolation scale	Subjective Loneliness	Social belonging and interpersonal well-being	4	0.61	2.01	0.95	−0.44
A	Alkan	2016	Turkey	509	22.36	The PSSM Scale (Goodenow, 1993) and UCLA Loneliness Scale (Russell et al., 1980; Russell, 1996)	Emotional Loneliness and Subjective Loneliness	School Membership	na	na	na	na	−0.21
A	Chen and Chung	2007	taiwan	319	na	Revised UCLA Loneliness Scale (R-UCLA) (Russell et al., 1980) and Social Connectedness Scale (SCS) (Lee et al., 2001)	Emotional Loneliness and Subjective Loneliness	Relational Belonging	74.29	11.84	39.97	8.58	−0.69
A	Satici, Uysal, and Deniz	2016	Turkey	325	20.96	UCLA Loneliness Scale (ULS-8; Hays and DiMatteo, 1987) and Social Connectedness Scale (Lee and Robbins, 1995)	Emotional Loneliness and Subjective Loneliness	Relational Belonging	15.86	7.33	12.84	3.54	−0.56
A	Hopmeyer and Medovoy	2017	USA	588	20.07	The UCLA Scale (Russell, 1996), and college belongingness (Asher & Weeks, 2014).	Emotional Loneliness and Subjective Loneliness	Relational and contextual (academic/social) forms of belonging	3.85	0.9	2.37	0.6	−0.55
A	Turton et al.	2018	Greece	281	22.28	The Social Connectedness Scale (SCS; Lee and Robbins, 1995) and the 20-item UCLA Loneliness Scale (Version 3) (Russell, 1996)	Emotional Loneliness and Subjective Loneliness	Relational Belonging	36.64	9.9	45.31	11.4	−0.83
A	Stice and Lavner	2019	USA	821	na	The UCLA Loneliness Scale (Russell, 1996), a 20-item self and the Cambridge Friendship Questionnaire (close friendship) (FQ; Baron-Cohen and Wheelwright, 2003).	Emotional Loneliness and Subjective Loneliness	Relational Belonging (Close-Personal)	na	na	na	na	−0.42
A	Seo	2020	South Korea	301	20.95	The revised UCLA loneliness scale (Russel, Peplau, & Cutrona, 1980) and the belonging subscale of the Interpersonal Support Evaluation List (ISEL) (Cohen et al., 1985)	Emotional Loneliness and Subjective Loneliness	Relational Belonging	11.94	2.36	40.16	10.23	−0.72
A	Wax et al.	2019	USA	660	20.37	The UCLA Loneliness Scale (Russell, 1996), and the 6-item College Belongingness Questionnaire (Asher & Weeks, 2010).	Emotional Loneliness and Subjective Loneliness	Relational Belonging and Institutional Belonging	3.77	0.82	2.14	0.54	−0.37
A	Nguyen, Werner, and Soenens	2019	Canada	220	18.54	The Belonging subscale from the Social Support Questionnaire (Sarason et al., 1987) and the UCLA Loneliness Scale (Russell et al., 1978).	Emotional Loneliness and Subjective Loneliness	Relational Belonging	5.49	1.09	2.43	1.14	−0.78
A	Ko et al.	2022	USA	518	19.24	The Social Connectedness Scale (Lee and Robbins, 1995) and the UCLA Loneliness Scale (Russell et al., 1978)	Emotional Loneliness and Subjective Loneliness	Relational Belonging	4.32	1.12	2.13	0.69	−0.77
A	Datu and Fincham	2022	Philippines andthe United States	1,189	19.84	The 8-item UCLA Loneliness Scale (Hays and DiMatteo, 1987) and Items in the relatedness subscale (*N =* 8) of the Basic Psychological Needs Satisfaction–General Scale (La Guardia et al., 2000)	Emotional Loneliness and Subjective Loneliness	Relational Belonging	na	na	na	na	−0.42
A	Brunsting et al.	2021	USA	126	na	The Three-Item Loneliness Scale (Hughes et al., 2004) and the six-item Student Belonging Scale (Dahill-Brown & Jayawickreme, 2016)	Emotional and social loneliness	Relational and institutional belonging	3.93	0.81	2.07	0.77	−0.39
A	Low, Bhar & Chen	2023	Australia,	138	21	The short form of the UCLA scale (Russell, 1996) and The Campus Connectedness Scale (CCS) (Lee & Davis, 2000).	Emotional Loneliness and Subjective Loneliness	Relational and institutional belonging	57.54	13.44	23.04	5.61	−0.51
B	Abebe et al.	2024	USA	2,782	18.2	The 15-item Social Connectedness Scale (Lee et al., 2008) and UCLA scale (Russell et al., 1980)	Emotional Loneliness and Subjective Loneliness	Relational Belonging	70.25	13.27	4.82	1.67	−0.49
B	Zou and Mu	2024	China	511	21.53	The Sense of Belonging Instrument (Hagerty and Patusky, 1995) and the Loneliness Scale (He, 2017)	Emotional and Social Loneliness	Emotional and Social Belonging	65.13	15.87	14.92	6.07	−0.19
B	Beloborodova et al.	2024	USA	648		The UCLA Scale (Russell, 1996) and the Sense of Social and Academic Fit Scale (Walton and Cohen, 2011)	Emotional Loneliness and Subjective Loneliness	Institutional/Academic and Relational Belonging	5.01	1.14)	1.67	(0.56	−0.41
A	Kusci, Oztosun, and Arli	2023	Turkey	284	21.63	The UCLA Loneliness Scale (Russell et al., 1978) and General Belongingness Scale (Malone et al., 2012)	Emotional Loneliness and Subjective Loneliness	Intrinsic and Relational Belonging	5.18	1.12	2.08	0.38	−0.626
B	Kochel, Bagwell and Abrash	2022	USA	517	19.52	The 10-item Loneliness in Context scale to assess feelings of loneliness in college (Asher & Weeks, 2014) and the 8-item ocial Connectedness Scale (Lee & Robbins, 1995).	Social and Situational Loneliness	Relational Belonging	3.85	1.17	2.77	0.87	−0.78
B	Ouzia, Wong and Dommett	2023	UK	241	21.53	The 12-item General Belongness Scale (GBS), Need to Belong Scale (NBS), (Malone et al., 2012), and the three-item UCLA Loneliness Scale (Hughes et al., 2004; Russell, 1996)	Emotional Loneliness and Subjective Loneliness	Intrinsic and Relational Belonging	5.22	0.99	5.52	1.81	−0.588
A	Mellinger et al.	2024	USA	360	29.88	The SBS: Sense of Belonging Scale (8 item version) (Hagerty and Patusky, 1995); the UCLA-Loneliness scale (Russell et al., 1980).	Emotional Loneliness and Subjective Loneliness	Intrinsic and Relational Belonging	3.33	1.08	2.24	0.69	−0.88
A	Morse et al.	2021	USA	234	19.89	The UCLA Loneliness Scale (Russell, 1996) the College Belongingness Questionnaire (Asher & Weeks, 2014).	Emotional Loneliness and Subjective Loneliness	Relational and Contextual Belonging	3.6	0.91	2.76	66	−0.54
A	Karababa and Tayli	2020	Turkey	613	na	The UCLA Loneliness Scale (Russell et al., 1978), and University Students Basic Needs Scale (USBNS) (Türkdoğan and Duru, 2012).	Emotional Loneliness and Subjective Loneliness	Institutional/Academic and Relational Belonging	16,88	na	13,17	na	−0.47
A	Blankenau et al.	2023	USA	692	19.87	The ten-item Loneliness in Context Questionnaire for College Students (Asher & Weeks, 2014), and the six-item College Belongingness Questionnaire (Asher & Weeks, 2014).	Situational or contextual experiences of loneliness	Relational and Contextual Belonging	3.72	0.91	2.31	71	−0.42
A	Worsley, Harrison, and Corcoran	2023	UK	904	19.4	The ULS-8 (Hays and DiMatteo, 1987), and a single item questionnaire “How strongly do you feel you belong to your accommodation.”	Emotional Loneliness and Subjective Loneliness	Contextual Belonging	2.77	0.86	18.91	5.55	−0.58
A	Knifsend	2020	USA	298	20.4	The four items university belonging survey (Anderman, 2002), and the UCLA Loneliness Scale (Russell et al., 1980).	Emotional Loneliness and Subjective Loneliness	Institutional/Academic and Relational Belonging	3.53	0.81	1.82	0.59	−0.17
B	Andreadis et al.	2023	Canada	356	na	An adapted version of the 5-item Perceived Cohesion Scale (Chin et al., 1999), and 10-item UCLA Loneliness Scale (Russell, 1996)	Emotional Loneliness and Subjective Loneliness	Relational and Group/Community Belonging	6.64	1.58	2.5	0.77	−0.35
A	Mounts	2004	USA	319	18.41	The 20-item revised UCLA (Russell et al., 1980), and Hurtado and Carter’s (1997) three-item belonging to campus scale.	Emotional Loneliness and Subjective Loneliness	Institutional and Relational Belonging	na	na	na	na	−0.37
B	Hansen-Brown et al.	2022	USA	348	19.67	The Student Belongingness, Engagement, and Self-Confidence Survey (Yorke, 2016), and the UCLA Loneliness Scale (Russell et al., 1978).	Emotional Loneliness and Subjective Loneliness	Institutional and Relational Belonging	21.37	3.78	45.91	16.09	−0.48
B	Brance et al.	2022	Greece	104	na	The social connectedness scale – revised (SCS-R; Lee et al., 2001), and the three items Loneliness Scale (Hughes et al., 2004).	Emotional Loneliness and Subjective Loneliness	Social and Relational Belonging	na	na	na	na	−0.43
B	Arslan, Yildirim, Zangeneh	2021	Turkey	315	21.65	The College Belongingness Questionnaire (CBQ; Arslan, 2021), and the Brief Adjustment Scale-6 (BASE; Cairns et al., 2020)	Emotional Loneliness	Relational, Institutional, and Emotional Belonging	51.93	10.45	na	na	−0.21
B	Hohne et al.	2022	Germany	496	22.94	The two items belonging uncertainty (Walton and Cohen, 2011), and the four items exclusion scale (Höhne and Zander, 2019)	Social Loneliness	Social and Relational Belonging	3.0	1.13	3.37	1.11	−0.271
B	Sebekova and Uhlarikova	2023	Slovakia	169	21.71	The Connectedness to School and Teacher (Waters and Cross, 2010), and the Student Stress Questionnaire (Pluut et al., 2015)	Social Loneliness	Relational, Institutional, and Social Belonging	15.76	2.88	na	na	−0.28
B	McFayden et al.	2023	USA	516	na	The Community belongingness scale (Virginia Tech Cook Counseling Center for the fall Cairns et al., 2020 semester), and Counseling center assessment of psychological symptoms (CCAPS; Locke et al., 2011)	Subjective Loneliness	Relational, Institutional, Emotional, and Social Belonging	3.06	0.96	na	na	−0.156
B	Werner et al.	2021	Germany	443	22.8	The three-item UCLA loneliness scale (Russell, 1996) and psychological support on a self-created scale (Werner et al., 2021)	Emotional Loneliness and Subjective Loneliness	Relational and Emotional Belonging	na	na	na	na	−0.58
B	Mäkiniemi, Oksanen, & Mäkikangas	2021	Finland	1,463	na	The Short Loneliness Scale (Hughes et al., 2004) and the sense of social belonging (Savolainen et al., 2018)	Emotional, and Social Loneliness	Relational, Emotional, and Social Belonging	5.29	1.01	na	na	−0.27
B	Marler et al.	2021	USA	238	19.7	The Sense of Belonging Scale (SBS; Hoffman et al., 2002) and the Depression Anxiety Stress Scale-Short Form (DASS; Lovibond and Lovibond, 1995)	Subjective Loneliness	Intrinsic and Relational Belonging	55.92	13.69	43.60	13.55	−0.39
B	Padmanabhanunni and Pretorius	2021	South Africa	337	21.95	The UCLA Loneliness Scale (Russell et al., 1980), satisfaction with Life Scale (Diener et al., 1985), and relational connectedness	Emotional and Subjective Loneliness	Relational, Emotional, Intrinsic, and Social Belonging	10.6	3.7	49.1	11.6	−0.81
B	Allan et al.	2021	USA	435	34.92	The 5-item NIH toolbox loneliness scale (Cyranowski et al., 2013), and Intolerance of uncertainty scale-12 (IUS-12; Carleton et al., 2007)	Emotional and Social Loneliness	Relational Belonging	na	na	na	na	−0.31
B	Graf and Bolling,	2024	USA	73	19.03	The UCLA Loneliness Scale (Russell et al., 1980), and The Social Connectedness Scale-Revised (Lee and Robbins, 1998)	Emotional and Subjective Loneliness	Relational Belonging	89.11	17.52	39.99	10.81	−0.65
B	Berman et al.	2022	USA	841	18.8	UCLA loneliness short scale (Hughes et., 2004) and the 9-item belongingness sub-scale from Interpersonal needs questionnaire (Van Orden et al., 2012).	Emotional Loneliness and Subjective Loneliness	Relational, Intrinsic, and Social Belonging	27.8	11.3	5.8	1.8	−0.186
B	Bonsaksen et al.	2022	Norway, USA, UK or Australia	354	na	The Loneliness Scale (De Jong-Gierveld and van Tilburg, 2006), The PsychoSocial Well-being (PSW) scale (Geirdal et al., 2021), and General Health Questionnaire 12 (GHQ-12) (Aalto et al., 2012)	Emotional and Social Loneliness	Relational and Social Belonging	3.2	2.7	4.6	2.9	−0.46
B	Weber et al.	2022	Germany	363	na	UCLA loneliness scale (Hays and DiMatteo, 1987), social support [brief form of perceived social support questionnaire (Kliem et al., 2015), quality of life (Diener et al., 1985)]	Emotional Loneliness and Subjective Loneliness	Relational Belonging	15.61	5.51	na	na	−0.3
B	Akkaya and Duy	2024	Turkey	498	na	Social and Emotional Loneliness Scale for Adults - Short Form (SELSA-S) by DiTommaso et al. (2004), and the Well Star Scale by Korkut-Owen et al. (2016)	Emotional and Social Loneliness	Relational Belonging	2.62	0.07	na	0.07	−0.56
A	Samadieh and Rezaei	2024	Iran	345	22.16	The Social and Emotional Loneliness Scale for Adults (SELSA-S), developed by DiTommaso et al. (2004) and the institutional integration scale (IIS), developed by Pascarella and Terenzini (1980)	Social and Emotional Loneliness	Relational, Institutional, Academic, and Social Belonging	19.13	3.773	11.29	4.707	−0.2

Group A includes studies with data collected before the COVID-19 pandemic (Before March 2020), while Group B comprises studies with data collected during (March 2020 – mid/late 2021) or after the pandemic (Late 2021 or early 2022 onward).

### 3.2 Overall average correlation between sense of belonging and loneliness

**Figure 3:**
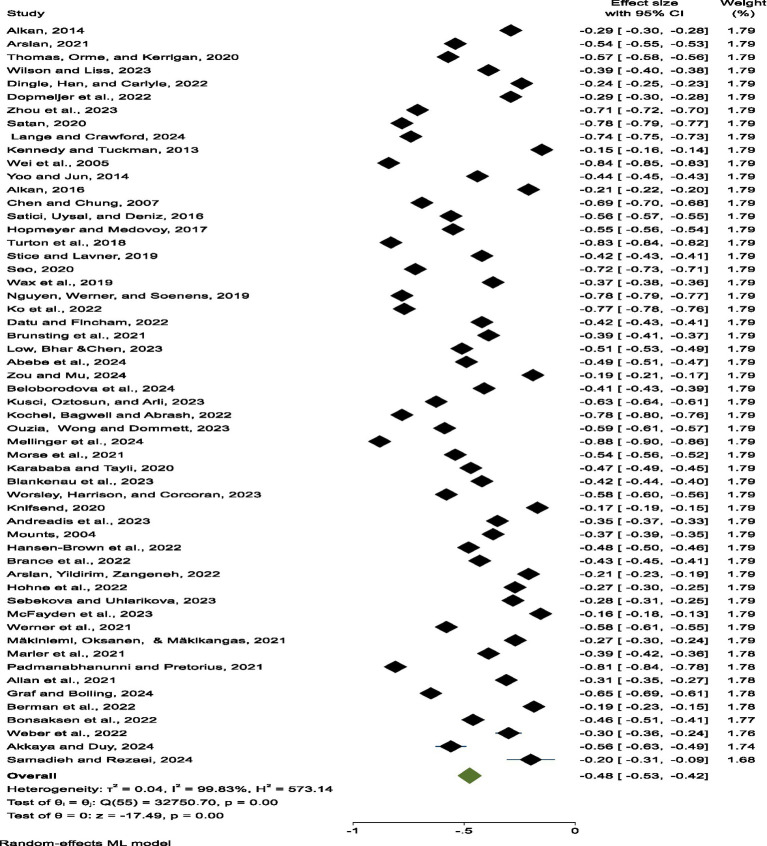
Forest plot of meta-analysis of published studies.

This metareview includes an exhaustive review of 56 independent studies with a large combined sample of around 30,062 individuals, applying a random effects model by deriving an overall ES (denoted *θ*) of −0.48 (see Figure 3). The 95% CI for the effect size is given as −0.529 to−0.422 and suggests a significant detrimental effect (z = −17.33, *p <* 0.001). This result is, in fact, meaningful as there is a strong relationship that is consistently reported across the examined studies, and the phenomenon under investigation is a strong predictor. The calculated confidence interval confirms this, as it does not fall on zero, providing evidence of a dependable pattern in the data. With heterogeneity inspection, this study shows a significant level of heterogeneity, as reflected by a large Q-statistic (Q = 32750.70, df = 56, *p <* 0.001) and a striking I^2^ value of 99.83%. This high I^2^ statistic indicates that a majority of the variance between studies is due to true differences in effect sizes, and not random error in sampling. Such a large heterogeneity can be interpreted in the sense that effects might not just vary with the populations of studies the effect size may be coming from (might be on account of several factors such as different condition measuring instrument types, data collection procedures, response rates, and researcher gender among others (Holzmeister et al., 2023; Nakagawa and Cuthill, 2007). Therefore, these reasons advocate for employing a random-effects model to better represent the variation in studies. In this meta-analysis, the weight given to each study seemed relatively equivalent, with every study contributing approximately between 1.7 and 1.8% to the total effect size. This moderate distribution of weights is interesting; it shows that a single study is not too heavy, which is also responsible for the robustness of the effect of −0.48. Given a substantial negative effect size and rather high heterogeneity, this latter underscores the urgent necessity to further explore subgroup moderators or sources of heterogeneity. Exploration of these moderating explanations may help to better understand the mechanisms behind the observed relation, which has been found in various contexts in several studies. Potentially, this analysis will also serve to shed light on the complexities and nuances of the relationship under study and to raise it to the general debate in the sector. The study also applied a leave-one-out analysis (i.e., iteratively removing studies to examine the stability of the pooled effect size; Shamim et al., 2023). Results of this analysis demonstrated a fairly consistent and robust pooled effect size, confirming the stability of the results and conclusions when individual studies were removed from the sample of studies. To examine publication bias, the analysis used Egger’s test (Egger et al., 1997) to test whether the funnel plot asymmetry was significant. A regression-based Egger’s test is used to assess small study effects by testing whether the coefficient significantly differs from zero. The estimated coefficient is 5.82, with a standard error of 3.35, a t-statistic of 1.74, corresponding *p*-value of 0.0882. This p-value is larger than 0.05, which suggests that there is no statistical evidence. Thereby, this result indicates a lack of small-study effects at the 5% level. A p-value close to 0.1, at least, shows a pattern which should be further explored for publication bias.

### 3.3 Moderator variables

#### 3.3.1 Calculating effect sizes

Adjusting for country, publication year, pre- and during/after the COVID-19 pandemic effects, the researcher explored three potential moderators (in a random model to explore the sources of heterogeneity). The meta-analysis found significant heterogeneity in effect sizes between countries, suggesting that the strength of the association varied across cultural groups and regions. For example, the combined effect size for Canada was −0.565 (95% CI: − 0.986 to − 0.144, *p* = 0.009), reflecting a significant moderate negative relationship. The USA, in contrast, had suboptimal comparison of −0.469 (95% CI: −0.562 to −0.375, *p <* 0.001), though still significant effect although less than the UK, and its association was less strong in specific countries: China, with an effect size of −0.450 (95% CI: −0.960 to 0.060, *p* = 0.083), and in which it did not reach statistical significance. The finding of non-significance in China suggests that the cultural influence or the potential moderators in the local context, such as cultural norms or specific stressors, may play as a moderating role in the relationship. Additionally, to determine the importance of these differences in effect sizes at the country level, a Q test of heterogeneity was performed, the results of which indicated a Q = 8881.15 (*p <* 0.001). This large p-value also suggests a large variability within individual countries that might lead to the existence of a country-specific relationship. This variability may be due to variation in social support structures and cultural values of community and individualism or the differential impact of the pandemic throughout regions. This finding underscores the notion that the relation itself varies as a function of regional context, and thus the possible need for more localised interventions in order to address such social or psychological concerns. The meta-regression analysis, with publication year as an important predictor variable, yields an R-squared value of 0.274. In other words, one could explain 27.4% of the variance displayed by effect sizes with the model, which is a moderate explanatory power. Looking at the effect sizes by publication year, we observe a sizable spread, with the points oscillating between −0.840 in 2005 and −0.370 in 2004. In addition, the Q-test was used to test heterogeneity among publication years, with a value of 7716.84 and *p <* 0.001. This strong statistical significance suggests extensive variability in effect sizes across years, suggesting the results could be dramatically moderated by the context of different years.

#### 3.3.2 Subgroup analysis

**Figure 4:**
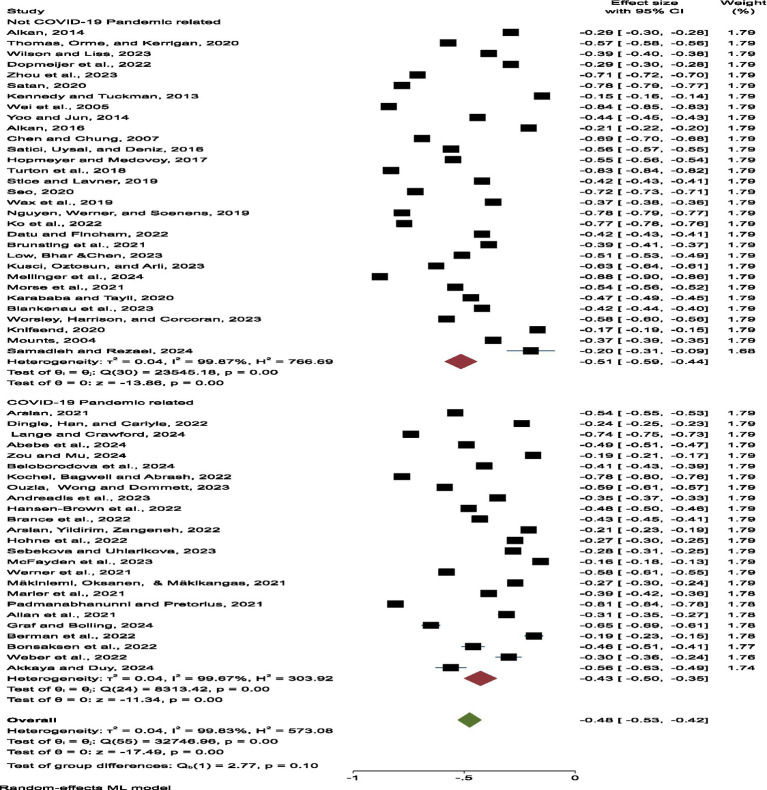
Forest plot of sub-group meta-analysis of published studies.

In the subgroup analysis of meta-analysis, this study sought to examine the effect sizes for two separate populations tested before the pandemic and tested during and after the pandemic comprehensively by using a random effect model and the Hedges’ method. This analysis aimed to investigate and compare the differences in effect sizes and dispersion between these two groups, Group A & Group B. Group A consists of population studies and has an average effect size of −0.515 (95% CI: −0.589 to −0.441, *p <* 0.001) and Group B has an average effect size that is slightly smaller at −0.427 (95% CI: −0.502 to −0.352, *p <* 0.001) (see Figure 4). Both sets of plots also show large negative correlations, indicating some consistency between the studies analysed. With regards to heterogeneity in Group A, a substantial amount of between-study variability (Q = 23549.70, *p <* 0.001) was found, with a tau-squared of 0.044, and I^2^ of 99.87%, indicating that almost all the variation can be attributed to true differences in effect sizes rather than sampling error. Group B exhibited a high degree of heterogeneity as well, with a Q-statistic of 8313.42 (*p <* 0.001), a tau-squared of 0.037, and an I^2^ of 99.68%. Such large heterogeneity within each group suggests a diverse range of effect sizes among the studies of both subgroups. The total heterogeneity summary of both groups was high, Q-statistic = 32750.70 (*p <* 0.001); tau-squared = 0.042; I^2^ = 99.83%. This implies widespread heterogeneity across the 56 studies, and a random effects model was employed. The test comparing the two group differences, Q = 2.66, was also not significant (*p* = 0.103), which indicates that the effect sizes were not significantly different between Group A and Group B. Consequently, on the one hand, there is a significant negative relationship in both subgroups, but, on the other hand, the strength of this relationship significantly did not vary between subgroups. This result implies that the general multimodal relationship found across studies is reliable across the variations represented by Group A and Group B, despite the variability.

## 4 Discussion

The aim of the present meta-analytic study was to systematically explore the associations between the experience of loneliness and the feeling of belonging in HE students before and during the COVID-19 pandemic. In particular, the researcher was interested to examine the direct associations between these two constructs as well as to investigate whether demographic and contextual factors such as the age and levels of education of participants and the geographical regions where the research is carried out might be associated with these constructs. Given the fact that the state of everyday social life and emotional wellbeing is reported to have changed decisively, not least following the onset of the COVID-19 pandemic, the researcher aimed to summarise and analyse the existing literature on this topic, both before and after the COVID-19 pandemic. For this aim, the researcher relied on a correlational methodology, compiling relevant cross-sectional studies from the literature. The design of these studies tends to have restricted the interpretation of the findings with respect to causality. The purpose of the meta-analysis is to synthesise these findings and conceptually to elucidate the complexity of the interplay between loneliness and belongingness among higher education students, contextualised in an era of shifting social developments.

This meta-analysis summarises 56 studies with 30,062 participants on the relationship of loneliness and belongingness in higher education students before and during the pandemic. The findings indicate a moderate to high negative association between these two constructs with an average effect size of (r = −0.48, 95% CI [−0.529, −0.422]). The strength of the negative relationship suggests an inversion such that degrees of loneliness are leading to marked reductions in feelings of belongingness across a variety of settings and samples. Narrow confidence intervals and a low standard error indicate that the estimate of this relationship is highly precise. Notwithstanding the consistency of this effect, a large heterogeneity existed among the included studies (Q = 32750.70, df = 56, *p <* 0.001, I^2^ = 99.83%), suggesting considerable variability in the magnitude of the effect sizes across studies. This substantial degree of heterogeneity indicates that the relationship between loneliness and belongingness is likely more or less moderate depending on the characteristics of study populations or methods. The high I^2^ demonstrates that nearly all of the variance of effect sizes is attributed to the true difference between the studies and not a random effect. This required a random-effects model to be used, which allows for differences between study populations and can be generalised to populations beyond the relatively narrow spectrum of the included studies. Moreover, the leave-one-out analysis also indicated the stability of the results. The pool effect size remained not significant by omission of any single study, suggesting a single study had no excessive impact on the overall result. The robustness of this result provides the confidence that the pooled estimate can be trusted and indicates that the negative association between loneliness and belongingness is generalisable across heterogeneous settings. The result of Egger’s test for publication bias is *p* = 0.0882, which indicates that there is no significant publication bias in studies included in this meta-analysis. A funnel plot was created for visual inspection of bias and its symmetry indicates that small, non-significant studies are not absent from the analysis. The lack of publication bias lends support to the validity of the findings, and it is unlikely that the effect sizes reported are inflated estimates of the association between loneliness and belongingness.

In the moderator analysis, geographical location was tested as a potential moderator in the moderator analysis to explore if geographical differences accounted for some of the heterogeneity in effect sizes. The sensitivity analysis indicates that the heterogeneity of the effect sizes exists to a great degree within the different levels of the moderators, especially country and publication year, corroborating the impact of contextual factors on the main relationship of the study. In particular, country-specific effect sizes differed substantially: Canada, moderate negative effect size (−0.565, 95% CI: −0.986 to −0.144, *p* = 0.009); USA smaller negative effect size of −0.469 (95% CI: −0.562 to −0.375, *p <* 0.001). The large Q-test for country differences (Q = 8881.15, *p <* 0.001) indicates substantial levels of heterogeneity across cultural and geographical settings, thereby highlighting the critical role of local social structures and norms in individual experiences and consequences. This finding is consistent with Bronfenbrenner’s (1979) ecological systems theory in which individual behaviour is considered to be situated within a multi-layered intertwined system of environmental influence, including cultural and social factors. Similarly, Gelfand et al. (2011), cultural tightness (the strength of social norms and the degree of tolerance for deviant behaviour) versus looseness can have profound effects on the way individuals perceive and relate to social structures, and could account for some of the observed differences described here. Regions with less tight social norms such as the U. S. may encourage more individualistic attitudes, and this orientation may influence the extent to which individuals use and are influenced by social support (Triandis, 1989; Hofstede, 2001). In contrast, collectivist cultures like in East Asia may be protected from a certain number of stressors compared to the individualist cultures due to stronger, more coherent social networks and community-oriented supports as illustrated by the non-significant effect size in China (−0.450, 95% CI: −0.960 to 0.060, *p* = 0.083). The attenuated associations in China may be due to the local cultural context, such as collectivism and family-oriented support structure, mitigating the effects of some of the individual-level variables (Pan et al., 2020). Moreover, Singh-Manoux and Marmot (2005) argue that social support systems vary greatly depending on the cultural context, and although society-based societies may provide an additional buffer to prevent people from being affected by their own stressors. For example, Japan’s collective, high-context culture, however, may be accompanied by implicit forms of social support which were less explicit, but still contribute to a protective buffer of social and emotional support (Kim et al., 2022). Therefore, variation in effect sizes between countries presented exhibited herein is likely to be indicative of these cross-cultural differences, with social norms pertaining to community, support and individualism providing the underpinning factors through which people experience and respond to social circumstances. Overall, these results further demonstrate the importance of taking cultural and regional differences into account in the interpretation of meta-analytic results, as they may impact not only the magnitude but also the direction of the relationships under study.

Analysis of subgroups by studies that were carried out before the COVID-19 pandemic (Group A) in comparison with those during and after the pandemic (Group B) yielded further information. Both proved to be negatively correlated significantly, albeit Group A (−0.515) had a stronger association than that observed within Group B (−0.427). This implies that the estimate was somewhat higher pre-pandemic but that the difference was not large enough to suggest a change in the nature of the relationship during or after the pandemic. Social isolation and loneliness were becoming increasingly prevalent as severe public health issues before the COVID-19 pandemic due to their negative impact on longevity and general health (Manchia et al., 2022; Özdemir and Tuncay, 2008; Pijpers, 2017; Vasileiou et al., 2019). These challenges have been further amplified by the pandemic and related public health responses (Kovacs et al., 2021; Patulny and Bower, 2022). While the strength of the statistical relationship has varied somewhat during or after the pandemic, the underlying dynamics of the relationship relatively unchanged, hence the pandemic is unlikely to have changed in the pattern or mechanisms of the relationship. The modest relation in the post-pandemic sample might suggest that even though loneliness endured, the processes underlying feelings of belongingness have changed, as a result of changes in types and practices of social interaction and in obeying the norms of social distancing. The second reason for this is that the patterns and drivers of this relationship (individual attributes, social processes or institutional arrangements) remain relatively stable, even though people are more lonely and the sense of belonging is more affected by the pandemic for some (Klinenberg and Leigh, 2024; Long et al., 2022). Also, if there’s any tendency that people may just temporarily adopt in their behaviour or perception in the COVID-19 pandemic, it may have become re-normalised over the time and gone back to the behaviour and perception before the pandemic. The observed short-period fluctuations could also be a transient and not the actual, long-term change in their relationship. Furthermore, for some individuals, there may have been less stigma or emotional dysphoria associated with being alone, which would have attenuated the connection between loneliness and not feeling that one belonged. The timing of data collection (i.e., early vs. late in the pandemic) may have affected the responses. Early lockdowns may have had greater psychological effects than later stages, once people had adjusted. For others, the pandemic led to individual growth, closer relationships with immediate family or a re-evaluation of what it means to belong. This change could have protected against the loneliness. Individuals adjusted to online communities, virtual meetups and digital support groups, all of which offered new avenues for belonging. These adjustments might have dampened the affective impact of loneliness for some people.

### 4.1 Limitations and future research

There are several important limitations to be noted despite the usefulness of this meta-analysis. First, it must be acknowledged that the present meta-analysis is essentially correlational. This is a limitation on generalisability that should cause us to treat the results not as definitive results about relationships among these variables, but as preliminary results. Further, although the initial criteria of inclusion were fulfilled by a number of studies, not all of them were included in the final analysis. Such exclusion was mainly due to incomplete reporting practices of the authors in the articles included in the systematic review, especially in relation to compliance with the APA style for reporting. Some studies did not give details of correlation matrices on their measured variables, while in some others, it was not clear whether or not the data had been used before. Another limitation lies with the potential of the current findings being affected by the few available samples addressing the relationship between feelings of belonging and other psychological factors. There were also relatively few observations in certain correlations overall. However, these figures are relatively small for us to generalise the results, and a small sample size can constrain the robustness of the findings among other populations. In addition, a number of studies employed less familiar or newly developed measures of constructs related to belonging or loneliness. The list included validities based on the combination of items from existing instruments. Although this lack of compatibility was not a common finding and would not have led to exclusions, it does highlight the potential variability and questionable reliability of the measures applied in this field of research. Another limiting factor is the lack of homogeneity of the characteristics of the study participants (who are, in all cases, higher education students) across the different studies. Because variability among the experiences of university students may mask the results and also impede the interpretation of the ways in which factors impact feelings of belonging and loneliness of students at different educational levels (pre and post pandemic eras).

As a sixth limitation, there was substantial heterogeneity between the studies included. Such a high degree of heterogeneity would indicate that the effect size that is reported in the various studies may be under the influence of a wide range of sample characteristics, measurement approaches, and contextual factors that are not sufficiently explored in this study. While some of this variability was addressed by using a random-effects model, much of the unexplained heterogeneity persists and can affect the overall precision and external validity of the results. Moreover, Egger’s test and the funnel plot indicated no substantial evidence of publication bias, we must be aware of the possibility of unobserved small-study effects impacting even the results. Publication bias testing assumes symmetry, and the absence of nonsignificant findings from unpublished results in the published literature may still influence the pooled effect size. This may lead to an inflated or deflated bias in the relationship between loneliness and belongingness. Another important limitation of the study concerns its use of correlation coefficients (r values), which limits the ability to make inferences about causality. The inverse association the researcher found between loneliness and belongingness is not necessarily a causal relationship, and other determinants, such as age, social support, and personality traits, could have had confounding effects not adjusted for in the present study. Although the moderator analyses the researcher carried out (including country, publication year and the comparison of pre- to post-pandemic periods) offered some informative insights, they ultimately translated into us having a limited comprehension of where the heterogeneity in effect sizes was coming from. Key potential moderators (e.g., cultural attitudes toward loneliness or different conceptualisations of belongingness) were not considered, but could significantly affect the results.

The data in the pre- versus post-pandemic subgroup analysis may not adequately reflect the complex and changing effects of the pandemic on social dynamics and mental health. Due to the rapid changes in the social and technological landscape, future research needs to employ longitudinal designs to examine how the association between loneliness and feelings of belongingness changes over time, particularly in a time of ongoing post-pandemic changes. This could lead to a greater understanding of these dynamics and better inform interventions to help individuals who are experiencing loneliness and a lack of belonging in multiple domains. In addition, future research may examine which technology and media/online connections mediate students’ sense of connection and belonging. As use of digital communication platforms is well-entrenched because of the COVID-19 pandemic, it is increasingly important for research to examine how these virtual spaces can either facilitate or act as barriers to the development of a community, especially for students who may be studying at a distance or are international students. Further comparative studies on higher education institutions that adopt different models—such as collegiate systems versus non-collegiate systems —could reveal important findings related to the impact of different structural models on feelings of belongingness and experiences of loneliness among students. The exploration of these differences may be useful for educational institutions interested in implementing structural reforms or policy changes that could enhance student wellbeing and promote a more caring education system. These results also have potential implications for programmes that seek to tackle loneliness by increasing feelings of belonging, particularly as the world transitions from the pandemic and social isolation remains a widespread issue. Furthermore, future research needs to investigate additional moderators beyond familiar factors like age, education, and region. Of particular interest would be examining how student populations with varying factors such as personality, social support networks and cultural beliefs of community engagement and isolation experience loneliness and belongingness. Being able to identify these subtle moderators may be crucial for designing specific interventions to promote the wellbeing and mental health of students. Furthermore, the value of supplementing quantitative studies with qualitative research methods as a means of more deeply understanding the lived experiences of loneliness and belongingness among students could be crucial. Conducting intensive interviews and focus groups would also enable students to articulate the complexities of their emotional landscapes, particularly in virtual learning environments. These could provide insight into what challenges and coping skills are uniquely experienced by students who interact with their classmates and instructors online.

## 5 Conclusion

The current meta-analysis provides evidence from a highly negative moderate to high association between loneliness and belongingness, a pattern that seems to be stable across populations and situational contexts. The large amount of heterogeneity between the studies underlines the necessity for more research to investigate other moderators which could account for the variation in effects. Country and publication year did not significant moderate the effect but the comparison between pre- and post-pandemic showed a small and significant difference in effect sizes, indicating that the social context of the pandemic affected the relationship between loneliness and belongingness. The absence of publication bias supports the credibility of these results, while the robustness of the summary effect size across studies highlights the generalisability of the negative association between loneliness and belongingness.

## Data Availability

The original contributions presented in the study are included in the article/supplementary material, further inquiries can be directed to the corresponding author.
